# Effect of H_1_- and H_2_-histamine receptor blockade on postexercise insulin sensitivity

**DOI:** 10.1002/phy2.33

**Published:** 2013-07-18

**Authors:** Thomas K Pellinger, Breanna R Dumke, John R Halliwill

**Affiliations:** Department of Human Physiology, University of OregonEugene, Oregon, 97403-1240

**Keywords:** Glucose, supply and distribution, oral glucose tolerance test, postexercise hypotension, skeletal muscle hyperaemia

## Abstract

Following a bout of dynamic exercise, humans experience sustained postexercise vasodilatation in the previously exercised skeletal muscle which is mediated by activation of histamine (H_1_ and H_2_) receptors. Skeletal muscle glucose uptake is also enhanced following dynamic exercise. Our aim was to determine if blunting the vasodilatation during recovery from exercise would have an adverse effect on blood glucose regulation. Thus, we tested the hypothesis that insulin sensitivity following exercise would be reduced with H_1_- and H_2_-receptor blockade versus control (no blockade). We studied 20 healthy young subjects (12 exercise; eight nonexercise sham) on randomized control and H_1_- and H_2_-receptor blockade (fexofenadine and ranitidine) days. Following 60 min of upright cycling at 60% VO_2 peak_ or nonexercise sham, subjects consumed an oral glucose tolerance beverage (1.0 g/kg). Blood glucose was determined from “arterialized” blood samples (heated hand vein). Postexercise whole-body insulin sensitivity (Matsuda insulin sensitivity index) was reduced 25% with H_1_- and H_2_-receptor blockade (*P* < 0.05), whereas insulin sensitivity was not affected by histamine receptor blockade in the sham trials. These results indicate that insulin sensitivity following exercise is blunted by H_1_- and H_2_-receptor blockade and suggest that postexercise H_1_- and H_2_-receptor–mediated skeletal muscle vasodilatation benefits glucose regulation in healthy humans.

## Introduction

During recovery from an acute bout of moderate-intensity dynamic exercise, humans experience a sustained rise in skeletal muscle blood flow from that of pre-exercise levels (Pricher et al. 2004; Halliwill et al. 2013). This sustained postexercise vasodilatation is found in both men and women (Senitko et al. 2002; Lynn et al. 2007), in both sedentary and endurance exercise trained individuals (Senitko et al. 2002; Lockwood et al. 2005; McCord and Halliwill 2006; McCord et al. 2006), and appears to be mediated by two (H_1_ and H_2_) histamine receptor subtypes (Lockwood et al. 2005; McCord and Halliwill 2006; McCord et al. 2006; Barrett-O'Keefe et al. 2013). Although the mechanisms of sustained postexercise vasodilatation are becoming more apparent, it remains unclear what purpose this increased blood flow might serve. Along these lines, prior investigations indicate that the time course of leg blood flow recovery does not match that of oxygen uptake, suggesting that skeletal muscle vasodilatation does not solely subserve oxygen delivery to the previously exercised skeletal muscle (Bangsbo and Hellsten 1998; Williams et al. 2005). The function, therefore, of this sustained postexercise histamine receptor–mediated vasodilatation has yet to be elucidated.

It is well established that skeletal muscle glucose uptake is enhanced during the first 90 min postexercise (Wasserman and Halseth 1998; Richter et al. 2001; Henriksen 2002) which corresponds to the peak glycogen synthesis rate in the previously exercised skeletal muscle (Bergstrom and Hultman 1966; Richter et al. 1989; Price et al. 1994; Casey et al. 2000). Several investigations in both animals (Grubb and Snarr 1977; Schultz et al. 1977) and humans (Hickner et al. 1991; Baron et al. 1994; Durham et al. 2003) suggest that increased limb blood flow enhances skeletal muscle glucose uptake. However, these prior studies have not examined the relationship between skeletal muscle blood flow and insulin sensitivity following exercise, as attenuation of this postexercise blood flow, and thus glucose delivery, may necessitate increased insulin secretion in response to a sustained elevation in blood glucose. Therefore, the current investigation was designed to determine if postexercise skeletal muscle vasodilatation influences glucose regulation sufficiently to impact insulin sensitivity following a bout of dynamic exercise. We tested the hypothesis that insulin sensitivity following exercise would be reduced in the presence of combined H_1_- and H_2_-receptor antagonists versus exercise alone (control condition).

## Methods

### Ethical approval

This study was approved by the Institutional Review Board of the University of Oregon. Each subject gave his or her informed, written consent prior to participation in the study. The study conformed to the principles of the Declaration of Helsinki.

### Subjects

Twenty healthy, nonsmoking, normotensive subjects (13 men; seven women), between the ages of 20 and 35, participated in this study. Twelve subjects (eight men; four women) participated in an exercise protocol and eight subjects (five men; three women) participated in a nonexercise sham protocol. For all study visits, subjects reported to the laboratory at least 3 h postprandial having refrained from alcohol consumption and exercise for 24 h and consumption of caffeine for 12 h. Subjects were taking no medications with the exception of oral contraceptives. In addition, female subjects were studied during the early follicular phase of their menstrual cycle or during the placebo phase of oral contraceptive use, to minimize the potential effects of reproductive hormones on cardiovascular and metabolic regulation.

### Screening visit

Subjects participating in the exercise protocol initially visited the laboratory to perform a peak aerobic power test on a cycle ergometer, in addition to self-reporting activity levels on two questionnaires. Subjects performed an incremental cycle exercise test (Lode Excaliber, Groningen, The Netherlands) comprised of 1-min workload increments to determine peak oxygen uptake (VO_2 peak_). Specifically, after a 2-min warm-up period of easy cycling (20–30 W), workload was increased by 20, 25, or 30 W every minute. Selection of the workload increment was based on self-reported subject activity levels, with the goal of producing exhaustion within 8–12 min. Whole-body oxygen uptake was measured via a mixing chamber (Parvomedics, Sandy, UT) integrated with a mass spectrometry system (Marquette MGA 1100, MA Tech Services, St. Louis, MO). Peak aerobic power was determined as either when subjects were unable to maintain 60 revolutions per minute, had obtained a respiratory exchange ratio of greater than 1.15, and/or had reached subjective exhaustion [rating of perceived exertion on the Borg (Borg 1970) scale of 19–20] within the 8- to 12-min period.

After resting for 10–15 min, subjects returned to the cycle ergometer to determine the workload corresponding to a steady-state oxygen consumption of 60% of VO_2 peak_. This workload was used on the exercise study days for the 60-min exercise bout, as this intensity and duration of exercise has been shown to evoke sustained (∼100 min) postexercise skeletal muscle vasodilatation (Pricher et al. 2004).

### Experimental protocol

For both the nonexercise sham and exercise protocols, subjects reported for parallel experiments on two separate days. The order of experiments was randomized between a combined H_1_- and H_2_-receptor antagonist (fexofenadine and ranitidine) and a control day. H_1_-receptor antagonism was produced via oral administration of 540 mg fexofenadine hydrochloride (Allegra; Aventis Pharmaceuticals Inc., Kansas City, MO). H_2_-receptor antagonism was produced via oral administration of 300 mg ranitidine hydrochloride (Zantac; Pfizer Consumer Healthcare, Morris Plains, NJ). Both drugs were orally administered immediately upon the subjects' arrival to the laboratory on the blockade day. These doses of oral fexofenadine (time-to-peak concentration ∼1.15 h and half-life ∼12 h) and ranitidine (time-to-peak concentration ∼2.2 h and half-life ∼2.6 h) have been shown to adequately block H_1_ and H_2_ receptors, respectively (Russell et al. 1998; McCord and Halliwill 2006). In addition, both fexofenadine and ranitidine are nonsedating, do not appear to cross the blood–brain barrier, or have any cardiovascular effects (Hardman and Gilman 2001).

On each study day, subjects were laid in the supine position for instrumentation. An intravenous catheter was inserted retrogradely in the dorsal vein of the left hand to obtain blood samples. During the exercise protocol, subjects underwent a 60-min period of seated upright cycling at 60% VO_2 peak_. Exercise of this intensity and duration produces a sustained (∼100 min) postexercise skeletal muscle vasodilatation (Pricher et al. 2004). During exercise, subjects consumed 10 mL of water per kilogram of body weight to offset volume loss during exercise. During the nonexercise sham study, the 60 min of cycling was replaced with 60 min of quiet rest.

Immediately after exercise or nonexercise sham, subjects consumed an oral glucose load to induce an increase in blood glucose concentrations. Each subject consumed 2.96 mL per kilogram of body weight (up to 296 mL maximum) of an oral glucose tolerance beverage (Trutol 100, NERL Diagnostics, East Providence, RI) that contained 338 mg of glucose per milliliter. Therefore, subjects received an oral glucose dose of 1 g per kilogram body weight up to 100 g maximum. Measurements were taken in the supine position prior to exercise or nonexercise sham and through 120 min following the oral glucose tolerance test (OGTT). Pre-exercise and postexercise measurements included heart rate, arterial pressure, femoral blood flow, as well as collection of blood samples for determination of oxygen saturation and concentrations of glucose, insulin, and C-peptide. During exercise, blood pressure and heart rate were measured every 15 min.

### Measurements

#### Heart rate and arterial pressure

Heart rate was monitored throughout both protocols via 5-lead electrocardiogram (Quinton Instruments, Bothell, WA). Arterial pressure was measured with an automated oscillometric device (Dinamap Pro100 vital signs monitor, Critikon Inc., Tampa, FL) during resting conditions. Arterial pressure during exercise was determined via manual auscultometry.

#### Leg blood flow

Mean blood velocities and diameters of the common femoral artery were measured using a linear ultrasound probe (10 MHz linear-array vascular probe, GE Vingmed System 5, Horton, Norway) placed distal to the inguinal ligament, approximately 2–3 cm proximal to the bifurcation. The entire width of the artery was insonated with an angle of 60 degrees and velocity measurements were taken immediately before diameter measurements. Leg blood flow was calculated as artery cross-sectional area multiplied by femoral mean blood velocity, doubled to represent both legs, and reported as mL min^−1^. Leg vascular conductance was calculated as flow for both legs/mean arterial pressure and expressed as mL min^−1^ mmHg^−1^.

#### Arterialized blood samples

To obtain “arterialized” blood samples, a heated hand vein was used (Morris et al. 1997). An intravenous catheter was inserted retrogradely into the dorsal vein of the left hand, which was then placed in a custom-made heating chamber (“hot-box”) that was flushed with air at 55°C. Skin temperature was raised to approximately 42°C, which is below the temperature that evokes sensations of pain. Arterialization of venous blood samples was confirmed by co-oximetry (i.e., saturation ≥ 97%; OSM-123, Radiometer Copenhagen, Denmark). Glucose concentrations of arterialized venous blood were measured in duplicate with a clinical glucose analyzer (YSI 2300 Stat Plus Glucose and Lactate Analyzer, YSI Life Sciences, Yellow Springs, OH). Blood samples were collected pre-exercise, half-way through exercise, immediately postexercise, and 10, 20, 30, 45, 60, 90, and 120 min following oral glucose load. Samples were promptly placed on ice, centrifuged at 4°C, separated, and stored at −80°C until analyzed.

### Blood hormone analysis

Analysis of insulin and C-peptide was conducted by the core lab at the Oregon Clinical and Translational Research Institute (OCTRI) by standard methods. The areas under the curve (AUC) for glucose, insulin, and C-peptide were calculated using the trapezoidal method. Estimation of whole-body insulin sensitivity was calculated from the glucose and insulin responses to the OGTT using the Matsuda insulin sensitivity index, which has a high correlation with indices of insulin sensitivity obtained from the euglycemic hyperinsulinemic clamp (Matsuda and DeFronzo 1999).

### Statistics

As our preliminary analysis did not indicate that sex had any effects on how subjects responded to the drug intervention, all subsequent statistical analyses were performed with men and women combined as a single group. Where pre-exercise data were available, Student's paired *t* test was conducted to test for baseline differences. Our primary analysis of data obtained postexercise was made using stepwise regression analyses to determine the effects of drug and time on recovery parameters using SAS Proc GLMSELECT (SAS v9.1; SAS Institute Inc., Cary, NC). A stepwise approach was used so that the possibility of both linear and quadratic regressions against time could be included in the analysis in addition to drug effects. The criterion for a term remaining in the model was set at 0.15, while significance was set at *P* ≤ 0.05. In addition to the primary analysis, we conducted a secondary analysis using repeated measures ANOVA using Proc Mixed with a priori contrasts of specific drug-time combinations. As such, we did not employ a multiple comparisons adjustment. All values are reported as means ± SEM unless otherwise noted.

## Results

### Subject characteristics

Subject characteristics are presented in Table 1. VO_2 peak_ values are within the normal range for young, healthy subjects of sedentary to endurance trained status.

**Table 1:** Subject characteristics

	Exercise protocol	Nonexercise sham protocol
N	12	8
Age (years)	24.3 ± 3.6	24.2 ± 5.3
Height (cm)	182.5 ± 12.1	179.4 ± 10.2
Weight (kg)	77.9 ± 18.7	79.3 ± 14.7
Body mass index (kg m^−2^)	23.1 ± 3.3	24.5 ± 3.5
VO_2 peak_ (mL kg^−1^ min^−1^)	54.0 ± 9.1	
Workload at 60% of VO_2 peak_ (watts)	180.5 ± 54.9	
Baecke sport index (arbitrary units)	12.3 ± 3.1	
Index of physical activity (MET h week)	168.7 ± 61.2	

Values are means ± SD. VO_2 peak_, peak oxygen consumption; MET, metabolic equivalents.

### Baseline

**Figure 1:**
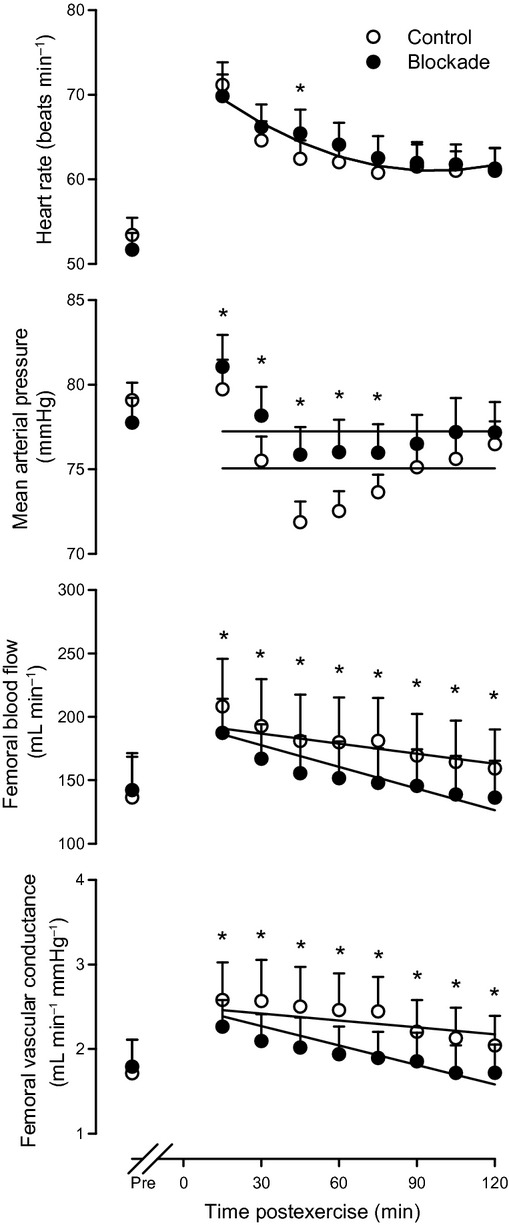
Hemodynamics before and after exercise. Heart rate (top panel), mean arterial pressure (upper-middle panel), femoral blood flow (lower-middle panel), and femoral vascular conductance (bottom panel) are shown prior to exercise (Pre) and through 2 h of recovery from a bout of dynamic exercise. Solitary regression lines for heart rate indicate the absence of main effects or interactions for blockade versus control during recovery from exercise. Parallel regression lines for mean arterial pressure indicate a main effect (*P* < 0.05 for drug effect) for blockade versus control during recovery from exercise. Nonparallel regression lines for femoral blood flow and femoral vascular conductance indicate an interaction (*P* < 0.05 for drug-time interaction) for blockade versus control across time during recovery from exercise. *n* = 12 for heart rate and mean arterial pressure and *n* = 8 for femoral blood flow and femoral vascular conductance. *P < 0.05 control versus blockade by repeated measures ANOVA. In this and subsequent figures, values are means ± SEM.

**Figure 2:**
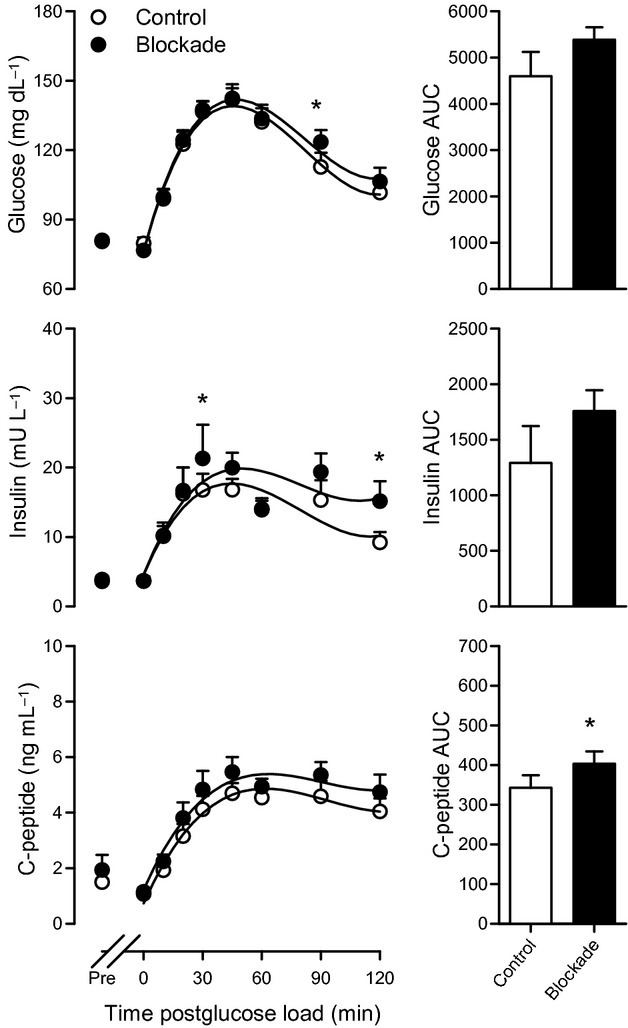
Blood and plasma concentrations before exercise and following postexercise oral glucose load. Blood glucose (top panel), plasma insulin (middle panel), and plasma C-peptide (bottom panel) are shown prior to exercise (Pre) and through 2 h following postexercise oral glucose load. Nonparallel regression lines for blood glucose and plasma insulin concentrations indicate an interaction (*P* < 0.05 for drug-time interaction) for blockade versus control across time during recovery from exercise. Parallel regression lines for plasma C-peptide concentrations indicate a main effect (*P* < 0.05 for drug effect) for blockade versus control during recovery from exercise. *n* = 12 for blood glucose and *n* = 11 for plasma insulin and C-peptide. *P < 0.05 control versus blockade by repeated measures ANOVA. AUC, area under the curve.

As shown in Figure 1, prior to exercise, supine resting heart rate was similar on both study days (control, 53.4 ± 2.0, vs. blockade, 51.7 ± 2.0 beats min^−1^; *P* > 0.05). Likewise, there were no differences in pre-exercise mean arterial pressure (control, 79.1 ± 1.1, vs. blockade, 77.8 ± 1.5 mmHg; *P* > 0.05), femoral blood flow (control, 136.3 ± 32.2, vs. blockade, 142.1 ± 29.4 mL min^−1^; *P* > 0.05), or femoral vascular conductance (control, 1.71 ± 0.4, vs. blockade, 1.79 ± 0.3 mL min^−1^ mmHg^−1^; *P* > 0.05). As shown in Figure 2, pre-exercise blood glucose was similar on both days (control, 80.6 ± 1.2, vs. blockade, 81.0 ± 1.6 mg dL^−1^; *P* > 0.05). Likewise, there were no differences in pre-exercise insulin (control, 3.6 ± 0.5, vs. blockade, 3.8 ± 0.6 mU L^−1^; *P* > 0.05) or C-peptide (control, 1.5 ± 0.1 vs. blockade, 1.9 ± 0.5 ng mL^−1^; *P* > 0.05).

**Figure 3:**
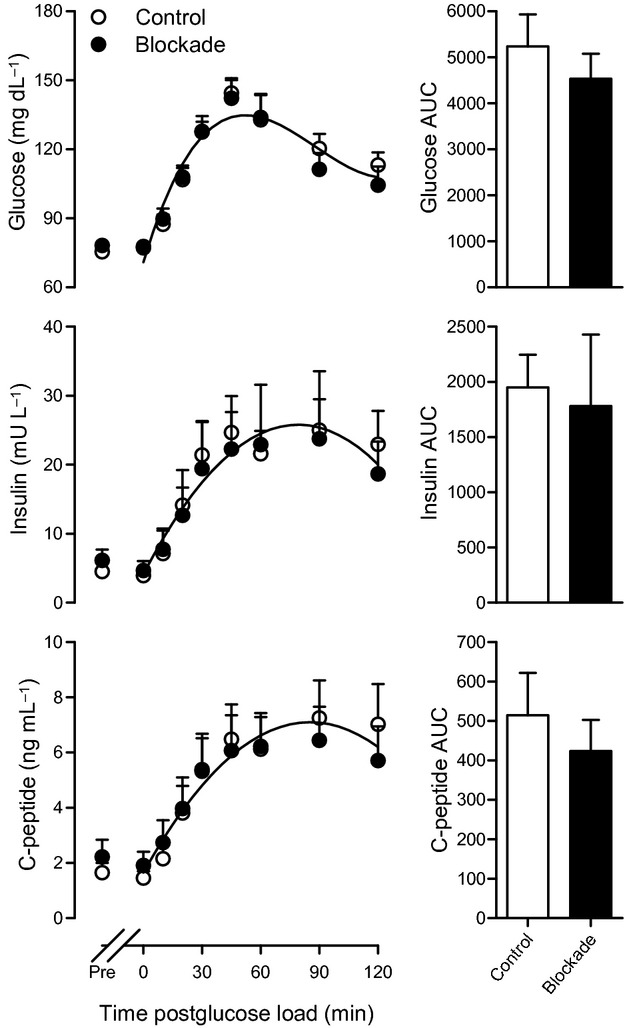
Blood and plasma concentrations before nonexercise sham and following postsham oral glucose load. Blood glucose (top panel), plasma insulin (middle panel), and plasma C-peptide (bottom panel) are shown prior to sham (Pre) and through 2 h following postsham oral glucose load. Solitary regression lines in each panel indicate the absence of main effects or interactions for blockade versus control during recovery from exercise. *n* = 8 for blood glucose and *n* = 7 for plasma insulin and C-peptide. AUC, area under the curve.

During the nonexercise sham protocol, presham resting heart rate (control, 53.8 ± 3.1, vs. blockade, 53.5 ± 3.6 beats min^−1^; *P* > 0.05) and mean arterial pressure (control, 76.5 ± 2.0, vs. blockade, 74.9 ± 1.7 mmHg; *P* > 0.05) did not differ between control and blockade days. As shown in Figure 3, presham blood glucose was similar on both days (control, 75.4 ± 1.7, vs. blockade, 78.2 ± 1.3 mg dL^−1^; *P* > 0.05). Likewise, there were no differences in presham insulin (control, 4.5 ± 1.3, vs. blockade, 6.1 ± 1.6 mU L^−1^; *P* > 0.05) or C-peptide (control, 1.6 ± 0.4, vs. blockade, 2.2 ± 0.6 ng mL^−1^; *P* > 0.05).

### Exercise

During the exercise protocol, the goal was to have subjects exercise for 60 min at 60% VO_2 peak_. The percentage of heart rate reserve (defined as maximal heart rate achieved during VO_2 peak_ testing minus the resting supine heart rate) attained during exercise (control, 70.2 ± 1.7%, vs. blockade, 69.8 ± 2.3%; *P* = 0.78) was consistent with the target workload. Heart rate increased from 53.1 ± 2.0 beats min^−1^ during supine rest to 150.8 ± 2.1 beats min^−1^ during exercise (measured 30 min into exercise bout) on the control day and from 51.2 ± 2.1 beats min^−1^ during rest to 149.4 ± 2.9 beats min^−1^ during exercise on the blockade day (*P* < 0.05 vs. rest on both days). Mean arterial pressure increased from 79.1 ± 1.1 mmHg during supine rest to 91.2 ± 1.9 mmHg during exercise on the control day and from 77.8 ± 1.5 mmHg during supine rest to 90.3 ± 1.7 mmHg during exercise on the blockade day (*P* < 0.05 vs. rest on both days). Blood glucose concentration during exercise was 76.6 ± 2.2 mg dL^−1^ on the control day and 75.0 ± 2.4 mg dL^−1^ on the blockade day (*P* > 0.05 vs. control). Plasma insulin concentration during exercise was 1.82 ± 0.1 μIU mL^−1^ on the control day and 2.33 ± 0.3 μIU mL^−1^ on the blockade day (*P* > 0.05 vs. control).

### Nonexercise sham

During the nonexercise sham period on both study days, neither heart rate (control, 53.3 ± 3.3, vs. blockade, 55.0 ± 3.6 beats min^−1^; both *P* > 0.05 vs. baseline) nor arterial pressure (control, 77.2 ± 2.7, vs. blockade, 76.2 ± 1.8; both *P* > 0.05 vs. baseline) changed from presham supine rest. Blood glucose concentration during the sham period was 77.2 ± 2.0 mg dL^−1^ on the control day and 80.0 ± 1.4 mg dL^−1^ on the blockade day (*P* > 0.05 vs. control). Plasma insulin concentration during the sham period was 4.61 ± 1.2 μIU mL^−1^ on the control day and 5.91 ± 1.7 μIU mL^−1^ on the blockade day (*P* > 0.05 vs. control). Previous research has shown that neither H_1_-receptor antagonism (Lockwood et al. 2005) nor H_2_-receptor antagonism (McCord et al. 2006) affects vascular conductance following nonexercise sham, thus, this study did not measure femoral vascular conductance following the nonexercise sham.

### Hemodynamics following exercise

As shown in Figure 1, heart rate following exercise declined similarly under both conditions (*P* < 0.05 for time effect). Similar to previous findings (McCord and Halliwill 2006), mean arterial pressure was higher following exercise on the blockade day compared with the control day (*P* < 0.05 for drug effect). The sustained elevation in both femoral blood flow and femoral vascular conductance during the control day was significantly blunted by the blockade, as both parameters declined more rapidly on the blockade day (both *P* < 0.05 for drug-time interaction). During the nonexercise sham protocol, postsham heart rate and mean arterial pressure did not differ between the control and blockade days (*P* > 0.05).

### Glucose, insulin, and C-peptide in response to oral glucose load

As shown in Figure 2, in response to oral glucose load, the rise in blood glucose concentration held higher values after the initial peak on the blockade day compared to the control day (*P* < 0.05 for drug-time interaction). Likewise, plasma insulin concentrations remained elevated longer on the blockade day than the control day (*P* < 0.05 for drug-time interaction). C-peptide concentrations were also greater on the blockade day than the control day throughout recovery from exercise (*P* < 0.05 for drug effect), resulting in a larger area under the curve.

As shown in Figure 3, during the nonexercise sham protocol, the glycemic response was similar on the control and blockade day, as blood glucose concentration peaked 45 min following oral glucose load on both days (*P* < 0.05 for time effect). Likewise, the rise in plasma insulin and C-peptide concentrations did not differ between the control and blockade day, both reaching their peak concentrations 90 min following the glucose load (both *P* < 0.05 for time effect).

**Figure 4:**
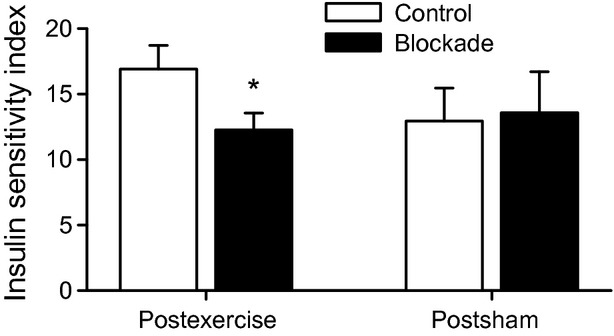
Matsuda insulin sensitivity index in response to oral glucose load following exercise and nonexercise sham. Open bars denote control day; filled bars denote H_1_- and H_2_-receptor blockade (fexofenadine and ranitidine) day. *n* = 6 for nonexercise sham protocol; *n* = 10 for exercise protocol. *P < 0.05 versus control.

The effect of H_1_- and H_2_-histamine receptor blockade on estimated whole-body insulin sensitivity, as indicated by the Matsuda insulin sensitivity index, is illustrated in Figure 4. The Matsuda insulin sensitivity index was reduced on the blockade day by 25 ± 8% compared to the control day (*P* < 0.05). Another way of expressing these results is that insulin sensitivity was 46 ± 14% higher in the presence of sustained postexercise vasodilation, when calculated from pre-exercise values. During the nonexercise sham protocol, there was no difference in the Matsuda insulin sensitivity index (*P* = 0.52).

## Discussion

The goal of this study was to determine the effect of the combination of orally administered H_1_- and H_2_-receptor antagonists on glucose regulation following a postexercise oral glucose load. In agreement with our hypothesis, the Matsuda insulin sensitivity index, an estimate of whole-body insulin sensitivity derived from glucose and insulin responses to an OGTT, was reduced following exercise in the H_1_- and H_2_-receptor blockade versus the control condition.

The finding that postexercise insulin sensitivity is reduced 25% during H_1_- and H_2_-receptor antagonism suggests delivery of glucose to skeletal muscle cells is blunted in the blockade condition, thus, requiring a greater secretion of insulin in response to a sustained elevation in blood glucose. It has been established that combined oral H_1_- and H_2_-receptor blockade attenuates postexercise vasodilatation in previously active skeletal muscles by approximately 80% (McCord and Halliwill 2006) which is in line with the postexercise vasodilatation found in this study. While the current investigation did not measure glucose uptake by the skeletal muscle cells per se, it indicates that glucose regulation is influenced by skeletal muscle vasodilatation following dynamic exercise.

### Determinants of insulin sensitivity following exercise

Recent studies employing muscle microdialysis have lent support to the notion that skeletal muscle vasodilatation aids in the movement of glucose from the central circulation to skeletal muscles following exercise. Pellinger et al. (2010) found that when postexercise skeletal muscle vasodilatation was blunted in humans via local H_1_-and H_2_-receptor blockade, interstitial glucose concentrations were attenuated. Similarly, Hamrin and colleagues (Hamrin et al. 2011) demonstrated increased tissue perfusion and skeletal muscle glucose uptake 12 h after the completion of a 2-h bout of one-legged cycling. Interestingly, this response was independent of enhancement of insulin responses, as they found similar increases in skeletal muscle glucose uptake in the postexercising and postresting legs in response to a hyperinsulinemic euglycemic clamp.

Emhoff et al. (2011) found that oral H_1_-and H_2_-receptor blockade reduced both femoral vascular conductance and leg glucose delivery following 60 min of cycling exercise. However, leg glucose uptake was not universally affected in recreationally active individuals. It is worth noting that the current investigation employed a postexercise glucose load, whereas the postexercise measurements in the Emhoff study were taken several hours postprandial. In addition, although glucose uptake was not consistently blunted by H_1_-and H_2_-receptor antagonism in the Emhoff study, they found a correlation between the effect of blockade on leg glucose uptake and subjects' absolute peak oxygen consumption, which suggests a significant histaminergic component to postexercise glucose regulation in individuals when muscle blood flow is most restricted or glucose metabolism most elevated.

Whereas the delivery of glucose itself is a potential key function of postexercise vasodilatation, perhaps equally important to the regulation of blood glucose is the delivery of insulin to the microvasculature, where it can impose both its hemodynamic and metabolic effects. Along these lines, physiological concentrations of insulin have been shown to evoke increased capillary recruitment (Coggins et al. 2001; Vincent et al. 2004; Emhoff et al. 2011) in humans. Movement of insulin from the central circulation to the skeletal muscle microvasculature and subsequently to the interstitium is required to initiate capillary recruitment and GLUT4 translocation, therefore, anything that aids insulin transport to the capillaries and interstitium should accelerate muscle glucose uptake. This notion is supported by a recent study by Chiu and coworkers who found that intramuscular injection of insulin resulted in an immediate rise in hindlimb glucose uptake in dogs (Chiu et al. 2008). Postexercise skeletal muscle vasodilatation likely facilitates insulin-mediated vasodilatation and capillary recruitment by enhancing delivery of insulin to the microvasculature when the stimulus for glucose uptake is high, such as following exercise. If this insulin delivery is attenuated by H_1_- and H_2_-receptor antagonists, that could help explain the reduced insulin sensitivity on the blockade days.

Another factor that may help explain reduced insulin sensitivity during H_1_- and H_2_-receptor blockade is the potential effect of H_1_- and H_2_-receptor antagonism on vascular permeability. If capillaries demonstrate a histaminergic increase in permeability following exercise, diffusion of glucose to the muscle cells would be expected to decline during H_1_- and H_2_-receptor blockade, resulting in greater recirculation of glucose. Along these lines, Thomas et al. (1995) found that activation of H_1_-receptors by histamine increased glucose uptake in cultured cardiac endothelial cells. Although a histaminergic effect on microvascular permeability (Majno and Palade 1961; Killackey et al. 1986; Hill et al. 1997; van Hinsbergh and van Nieuw Amerongen 2002) has been observed in postcapillary venules (Svensjo and Grega 1986; van Hinsbergh and van Nieuw Amerongen 2002) and in studies on cultured human umbilical vein endothelial cells (Niimi et al. 1992; Ikeda et al. 1999), there is a dearth of evidence that H_1_ receptors mediate increases in permeability of the in vivo skeletal muscle vascular endothelium. Therefore, while it is feasible that histaminergic effects on capillary permeability factor into reduced postexercise insulin sensitivity during H_1_- and H_2_-receptor blockade, there is currently limited evidence to support this notion.

A fourth possible explanation of the results of the current investigation is that orally administered H_1_- and H_2_-receptor antagonists following exercise may inhibit skeletal muscle cell glucose transporters. Blunted glucose transport would decrease the concentration gradient between the capillaries and the interstitial space, possibly reducing diffusion of glucose and leading to the sustained elevation of blood glucose and insulin secretion in response to oral glucose load. As previously noted, although histamine has been shown to stimulate glucose transport in cultured cardiac endothelial cells in an H_1_-receptor–dependent manner, this result was attributed to effects on endothelial permeability, as opposed to factors such as enhanced GLUT4 translocation (Thomas et al. 1995). We were unable to find compelling evidence of a histaminergic effect on skeletal muscle glucose transport or GLUT4 activity, so it is unlikely that this is a mechanism that plays a role in our investigation.

If pancreatic insulin secretion was altered by H_1_- and H_2_-receptor antagonists, this could help explain the prolonged elevation in blood glucose and reduced insulin sensitivity following postexercise oral glucose load. However, insulin release in response to intravenous glucose administration has been shown to be unaltered by H_1_- and H_2_-receptor antagonists (Pontiroli et al. 1982), and H_2_-receptor blockade had no effect on insulin secretion following oral glucose tolerance tests (Scarpignato et al. 1981). As seen in Figure 2, the plasma insulin and c-peptide responses to postexercise oral glucose load in this study were equal or greater on the blockade day, which was likely in response to reduced glucose delivery during the H_1_- and H_2_-receptor blockade.

An effect of H_1_- and H_2_-receptor antagonism on hepatic glucose uptake or release could contribute to reduced postexercise insulin sensitivity during H_1_- and H_2_-receptor blockade. However, we were unable to find evidence in the literature of reduced hepatic glucose uptake or enhanced hepatic production during H_1_- and H_2_-receptor blockade. Moreover, the combination of postexercise sympathetic withdrawal (Halliwill et al. 1996) and consumption of an oral glucose beverage should suppress gluconeogenesis and glycogenolysis, likely minimizing any impact of H_1_- and H_2_-receptor blockade in this scenario.

Finally, an impact of H_1_- and H_2_-receptor blockade on gastric emptying could help explain the results of this study. Previous investigations on the effect of oral H_2_-receptor antagonists on gastric emptying have yielded equivocal results, as H_2_-receptor blockade has been found to facilitate (Ohira et al. 1993) and delay (Forrest et al. 1976) gastric emptying in humans. However, in the current investigation, the nonexercise sham protocol was undertaken to examine the effect of H_1_- and H_2_-receptor blockade on insulin sensitivity in the absence of prior exercise. As illustrated in Figure 3, we found no difference in the glycemic, insulin, or c-peptide response between the control and blockade days during the nonexercise sham protocol. Combined with the nearly superimposable rise in blood glucose in response to the postexercise oral glucose load, this indicates that any effects of H_1_- and H_2_-receptor blockade on gastric emptying in our study were minimal.

### Methodological considerations

Although the oral glucose tolerance test (OGTT) has been widely used to assess glucose tolerance in a wide array of populations (Cobelli et al. 2007), numerous factors associated with the OGTT may affect its reproducibility. These factors include variations in glucose absorption, neurohormonal interactions, splanchnic glucose uptake, and possible effects on incretin hormones, which are known to stimulate insulin secretion in response to glucose ingestion (Muniyappa et al. 2008). However, when combined with various indices of insulin sensitivity, such as the Matsuda insulin sensitivity index, the OGTT can be used to estimate β-cell function and insulin sensitivity in humans. Furthermore, significant advantages of OGTT include its more physiological delivery of glucose, its relative ease of use, and reduced invasiveness relative to the FSIVGT and euglycemic hyperinsulinemic clamp. Finally, as previously indicated, the results of our nonexercise sham protocol suggest that the combined H_1_- and H_2_-receptor blockade had no discernible effect on the glycemic response to OGTT in the absence of previous exercise.

As previously noted, oral administration of H_1_- and H_2_-receptor antagonists could, hypothetically, be working through mechanisms other than the peripheral vascular effects we have studied. As such, local blockade in the skeletal muscle circulation may have provided a more select, albeit more invasive experimental paradigm for the study of vascular effects. However, given the widespread use of fexofenadine and ranitidine to treat allergies and gastrointestinal disorders, using oral blockade in this study has more relevance to real-world circumstances than a study relying on local blockade.

### Perspectives

The results of the current investigation indicate that commonly used medications may impact normal glucose regulation following exercise. Although the current investigation was conducted on healthy subjects, these findings reveal potential implications of H_1_- and H_2_-receptor antagonists for those who depend on exercise to regulate their blood glucose levels. This may be particularly relevant to populations which have reduced skeletal muscle perfusion, such as the aged, obese individuals, and those suffering from Type 2 diabetes mellitus. For example, it is possible that by mediating glucose regulation, postexercise vasodilatation may be an important mechanism by which to circumvent insulin resistance in diabetes. Furthermore, effects of these widespread medications may confound exercise as a “treatment” for both the healthy population and those suffering from various diseases. Further research is warranted to investigate the mechanisms behind this apparent relationship between cardiovascular and metabolic regulation, including studies to determine if these findings extend to those with disease, such as those suffering from diabetes.

In conclusion, H_1_- and H_2_-receptor blockade reduced postexercise whole-body insulin sensitivity by 25%, as estimated by the Matsuda insulin sensitivity index. These findings suggest a histaminergic effect on postexercise glucose regulation in healthy humans.
